# Trueness of implant placement, safety and surgeon experience with dynamic computer-assisted implant surgery : a prospective multi-centre cohort study

**DOI:** 10.1186/s40729-026-00698-y

**Published:** 2026-06-26

**Authors:** Valentin Herber, Michael Gahlert, Achim von Bomhard, Florian Stelzle, Marcel Hanisch, Sebastian Kühl, Stefan Roehling

**Affiliations:** 1 Department of Oral Surgery, University Center for Dental Medicine Basel UZB, University of Basel, Basel, Switzerland; 2 Private Dental Clinic “Oralchirurgie T1”, Munich, Germany; 3 Clinic for Oral and Cranio-Maxillofacial Surgery, Swiss MAM Research Group, Department of Biomedical Engineering, University Hospital Basel, University of Basel, Basel, Switzerland; 4 Private Dental Clinic, “INN TAL MKG”, Rosenheim, Germany; 5 Private Dental Clinic “Jaws and Faces”, Höhenkirchen, Germany; 6 Department of Cranio-Maxillofacial Surgery, University Hospital Muenster, Muenster, Germany

**Keywords:** Accuracy, Clinical decision-making, Dental implants, Cone-beam computed tomography, Dynamic navigation

## Abstract

**Purpose:**

This prospective multi-centre cohort study aims to evaluate the trueness and safety of implant placement using a Dynamic Navigation System (DNS) for implant bed preparation.

**Materials and methods:**

A total of 109 dental implants were inserted using a DNS for implant bed preparation in 76 patients by five experienced surgeons in four study centres. Before implantation, a preoperative CBCT scan was performed for each patient. This was subsequently aligned with an intraoral scan. After digital planning of the implant position, a holding tray for the intra-oral marker was designed and printed with a 3D printer. Procedure duration as well as adverse events were recorded. After implant bed preparation with DNS, the implants were placed in a freehand manner. Postoperative surface scans were superimposed onto the pre-operative planning data to evaluate the angular and spatial deviations. Descriptive statistics were followed by a one-sided, one-sample t-test to compare the data with performance goals extracted from literature-based benchmark data from freehand surgery.

**Results:**

The overall mean angular deviation was 3.37°±2.01° with a minimum of 0.20° and a maximum of 11.10°. The mean mesiodistal and orovestibular deviation at implant tip was 1.18 ± 0.66 mm. 3D deviation at implant base showed a mean ± SD of 1.24 ± 0.65 mm. No Serious Adverse Device Effects (SADE) were reported. The angular and mesiodistal, and orovestibular deviations were significantly better than the performance goals from freehand surgery (*P*<.001). The mean VAS was of 87.5 ± 14.3.

**Conclusions:**

The study-related DNS demonstrates trueness, safety and surgeon satisfaction.

**Supplementary Information:**

The online version contains supplementary material available at https://doi.org/10.1186/s40729-026-00698-y.

## Background

Implant-supported restorations have emerged as a standard and widely implemented therapeutic approach for the rehabilitation of edentulous sites [1]. Implant placement must match the precision of the prosthetic planning to ensure the long-term survival and success of both the implant and the restoration. Accurate positioning of dental implants is critical, as improper placement can result in compromised esthetics, reduced functional outcomes, increased risk of infection, or even failure of the restoration [2].

Over the past several decades, various computer-assisted technologies have been developed to enhance the accuracy and predictability of precise implant placement. Firstly, Static Computer-Assisted Implant Surgery (s-CAIS) combining Cone-Beam Computed Tomography (CBCT) scans, Intraoral Scans (IOS), and Computer-Aided Design/Computer-Aided Manufacturing (CAD/CAM) of surgical templates enables highly precise implant placement [3]. The use of a surgical template during implant surgery exhibits reliable and accurate outcomes [4]. Some risks associated with CAIS have been highlighted, including deviations in implant positioning caused by errors in digital planning, inaccuracies in surgical guide fabrication, or misalignment during surgery, as well as potential damage to critical anatomical structures due to improper guide placement [5]. The use of a surgical guide can compromise visualization of the surgical site and limit the delivery of irrigation to the underlying bone, thereby increasing the risk of thermal elevation during osteotomy preparation [6].

Dynamic Computer-Assisted Implant Surgery (d-CAIS) has emerged to provide real-time guidance and intraoperative flexibility, with enhanced visualisation of the surgical field. This approach is based on real-time tracking of drills by utilizing a miniaturized intraoral marker mounted on a tray and a camera mounted on a handpiece, integrating an implant planning with the 3D preoperative virtual plan derived from CBCT imaging and intraoral scanning [7]. Dynamic Navigation Systems (DNS) offer significant potential to enhance dental implant surgery, and d-CAIS exhibits optimal clinical results in terms of trueness. In fact, previous studies have reported high accuracy and reliable clinical outcomes with DNS, with several investigations demonstrating comparable or improved accuracy relative to freehand or static guided implant placement approaches [8–13]. Several DNS are currently available. Wang et al. summarized the current principles and clinical applications of d-CAIS and highlighted the ongoing need for further research focusing on the accuracy of d-CAIS, as well as on complications and surgical time [14]. The study-related DNS was already investigated in vitro [7, 15, 16] and in vivo [9, 17, 18]. However, most available clinical studies are single-centre investigations with limited data on reproducibility across different surgeons. Therefore, further prospective multi-centre clinical studies are needed to better evaluate the generalizability and clinical applicability of DNS. Furthermore, the available data on the clinical performance, trueness, and safety of DNS remain limited. The aim of this prospective multi-centre clinical study is to evaluate the trueness, safety and surgeon experience of implant placement using a study-related DNS compared to benchmark data from freehand surgery. Trueness, defined by the closeness of the actual implant position to the planned one is assessed based on deviations in implant positioning, specifically the angular deviation and the mean deviation at the implant base and tip in the mesiodistal and orovestibular direction [19]. Additionally, the study aims to confirm the safety of the study-related DNS by collecting the proportion of patients experiencing Serious Adverse Device Effects (SADE).

It is hypothesized that d-CAIS achieves greater trueness than freehand surgery and outperforms benchmark data from freehand procedures in terms of trueness.

## Materials and methods

### Study population

This study was conducted as a prospective, multi-centric clinical trial for the implant rehabilitation of partially edentulous patients using d-CAIS. The trial was carried out across four study centres in Germany. The study was approved by the Ethics Committee of Bayerische Landesärztekammer (Reference Number: 21053), registered in the DRKS (Deutsches Register Klinischer Studien) under the reference DRKS00030849 and was conducted in accordance with Good Clinical Practice standards and the Declaration of Helsinki.

The inclusion criteria were subjects aged 18 years or older who had provided informed and written consent, partially dentate patients (mandible or maxilla) indicated for one or more dental implants with sufficient teeth in the arch for tray (marker fixation) registration, sufficient peri-implant soft tissue volume, and adequate horizontal and vertical bone for the placement of implants with a minimum length of 6 mm and a width of 3.3 mm. Additionally, patient acceptance, as well as maintenance, was mandatory. Patients with any of the following exclusion criteria were not allowed to participate: (i) lack of compliance or failure to provide consent, (ii) mental illness and/or depression or vulnerable group of the population (e.g., prisoners, serious drug addicts, those with developmental problems) which hampers their ability to provide the declaration of informed consent, (iii) general contraindications to implant treatment, (vi) need for bone and soft tissue augmentation procedures.

The primary objective of this study was to evaluate the effectiveness of implant bed preparation using the study-related DNS compared to benchmark data from freehand surgery. Effectiveness was assessed based on deviations in implant positioning, specifically the angular deviation from the planned Z-direction and the mean deviation at the implant base and tip in the XY-direction.

### Sample size calculation

The sample size was calculated by an independent statistician. Sample size estimation was based on data reported by Edelmann et al. [9], for which raw data were available and subjected to square root transformation to stabilize variance. The sample size calculation was based on XY-deviation measured on implant base. Assuming a power of 80% and a two-sided alpha level of 0.025, the required sample size was 63 patients and 82 implants. 5% operational dropouts were expected.

### Clinical procedures

Implant placements were performed by five experienced surgeons across four study centres in Germany, only one of which had previous experience with DNS. All surgeons were part of the research team. Before the start of the study, all participating surgeons underwent standardized training in the use of DNS. The study was divided into two phases. In the first phase, referred to here as operation initialized phase, the patient was examined by the surgeon and prepared for the surgery, but there was still a decision to be made by the surgeon whether to proceed with the operation or not. In the second phase, referred to as surgery began phase, the surgeon decided and performed the surgery.

Before implant placement, a preoperative CBCT and an intraoral surface scan were obtained for each patient. The registration (matching) of the CBCT and surface scan data was performed centrally for all study centres by a qualified expert using dedicated planning software (coDiagnostiX, Dental Wings GmbH, Chemnitz, Germany). The type, length, and diameter of the dental implants were determined by the responsible surgeons at each study centre based on clinical and anatomical considerations. A surgical tray was designed using the coDiagnostiX planning software, and the printing of the trays for all study centres was carried out by a certified and qualified central facility. The planning data were converted into the Generic Planning Objects Container (.genxa) file format using an integrated software tool within the planning software and were subsequently exported to the DNS (DENACAM Navigation system, MiniNavident, Liestal, Switzerland) via Universal Serial Bus (USB) transfer.

Only the implant bed preparation was done with the study-related DNS. Most of the implant bed preparation with the DNS was performed under local anesthetic solely (97,26%). One patient was operated under additional- general anaesthesia, and another one was operated under analgosedation. During the surgery, osteotomy preparation using different drills were performed according to the manufacturer’s instructions. Insertion torque was measured using a torque control device. The duration of the whole surgery including bur registration was recorded. The implants were placed in a freehand manner.

Intraoperative, and immediate postoperative complications including Adverse Events (AE), Serious Adverse Events (SAE), and their potential connection to the study device were reported.

### Primary Outcome Measure: trueness measurement procedure

After the implant placement procedure, compatible scanbodies were connected to the implants and postoperative surface scans were generated for each patient. The scans (STL) were imported to the planning software and automatically aligned with the preoperative data. The integrated treatment evaluation tool within the software was employed to automatically quantify the angular and spatial deviations between the planned and actual implant positions at implant tip (apex) and base (shoulder) (coDiagnostiX Version 10.8.1.1039, Dental Wings GmbH, Chemnitz, Germany). These data were transferred to an Excel sheet (Microsoft Excel 16.0; Microsoft Corp., Redmond, USA) for further statistical analysis.

### Surgeon satisfaction measurement

The investigator’s satisfaction with the test product was measured using the Visual Analog Scale (VAS), where 0 mm represents an unsatisfactory outcome, and 100 mm represents a satisfactory outcome for the surgeon. Surgeon satisfaction questionnaires were collected in a standardized and anonymized manner directly after the surgical procedures.

### Performance evaluation

The objective performance goals (δ) were calculated during the planning of the clinical study (in 2022) from results of a comprehensive literature research on clinical studies reporting deviation of freehand implantations. This literature research was done by two independent investigators during the planification of this clinical trial using PubMed^®^. The inclusion criteria were clinical trials including patients above 18 who are partially edentulous, publications showing measurements of differences between planned and real position of dental implants using the freehand method, both clinical data from prospective and retrospective in vivo study designs, metanalyses, systematic reviews, guidelines, consensus articles and editorials were considered. In vitro data, case reports, duplicates and reviews, as well as articles with no data related to the research topics, publications where full text was not available, publications not written in English or german were not considered. Out of a total of 26 publications identified, 11 were excluded as irrelevant based on abstract screening. After full-text evaluation and based on appraisal criteria including methodology, appropriate patient population (partially edentulous patients), study quality, and clinical relevance, 8 publications were excluded, resulting in 7 publications deemed relevant and included in the final analysis [20–26]. All articles were not older than 10 years and the range of publication was between 2012 and 2020.

The mean deviations were obtained as weighed means taking into account the reported standard deviations and sample sizes.

The resulted performance goals were defined as following:

(δ) (Angle) = 3.5° (95%-CI: 3.1–3.9°]

(δ) (XY-deviation, tip) = 2.3 mm (95%-CI: 1.9–2.6 mm]

(δ) (XY-deviation, base) = 1.2 mm (95%-CI: 1.0–1.4 mm]

The upper limits of confidence intervals (3.9°, 2.6 mm and 1.4 mm, for the angular, mesiodistal and orovestibular deviations at implant tip and base, respectively) were defined as performance goals. Technically transformed values (square-root-transformation) were used in the statistical analysis since the distribution of distances should not relevantly deviate from normal distribution.

### Statistical analysis

The sample size analysis as well as the statistical analysis was performed by an independent statistician. Mean of absolute values and the 95% confidence intervals were calculated for angular and spatial deviation (mesiodistal and orovestibular, apicocoronal, 3D deviation at the implants tip and base, respectively). Regarding the performance analysis, the null hypothesis was defined as: H_0_: Δ(γ) ≥ δ_γ_, where γ represents the predefined deviation (angular, mesiodistal and orovestibular deviations at implant base or tip), Δ represents the mean deviation detailed as the mean of paired differences (realized vs. planned value), δ_γ_ represents the performance goal. To meet the assumptions of parametric testing and given the skewness of the data, the data were subjected to a square root transformation, resulting in a distribution that did not deviate significantly from normality. A one-sided, one-sample *t*-test was then applied to evaluate on Δ(γ) against δ_γ_ using a significance level of α = 0.025. In addition, Δ(γ) was reported together with its one-sided 95% upper confidence limit.

## Results

### Patients’ information

Between January 2023 and October 2024, a total of 76 patients meeting the inclusion and exclusion criteria were enrolled in the study, and 109 dental implants were placed. All subjects understood and signed the informed consent. All subjects were partially edentulous, were ≥ 18 years of age, and required one or more dental implants as part of their treatment. According to the treating investigators, all enrolled subjects were suitable to get the implant with sufficient soft and hard tissue condition, and occlusion. All subjects could bear the risks of implant placement and were not enrolled in any other study. A total of 109 implants were planned in these 76 subjects. The patient’s age was 52.3 ± 15.88 years. Of the total patient population, 31 were males (40.79%) and 45 were females (59.21%).

**Fig. 1:**
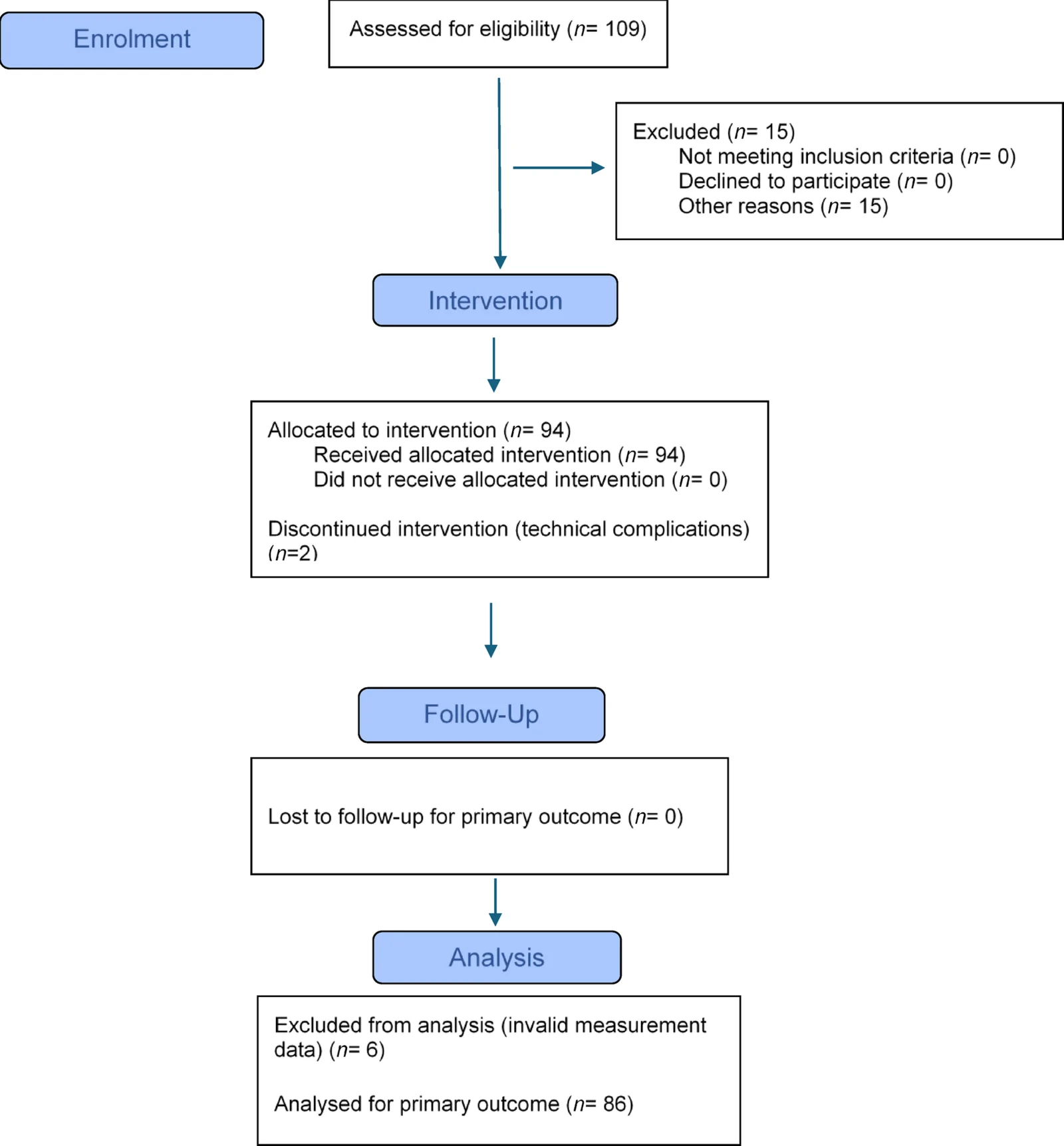
STROBE flow chart of the study (n = number of implants). Intervention and follow-up were conducted in the same visit

Three patients (with 8 implants) dropped out in the operation initialized phase. Another six patients (with 7 implants) were excluded before operation began phase based on the investigators’ decisions. Contributing factors included poor tray fit, unavailability of planning data at the start of surgery, delayed implant delivery, interference between the contra-angle handpiece and the DNS camera, restricted mouth opening, and missing calibration of the implant motor. Two patients (each with 2 implants) dropped out after surgery due to procedure-related issues. In one case, primary stability could not be achieved with the planned implant diameter, and a wider implant had to be placed instead. In the other case, the tray holder did not achieve a stable and defined position in the patient’s mouth, which would have prevented accurate transfer of the planned implant position. Another three patients (with 6 implants) were excluded because of missing or invalid measurement data. Consequently, the final analysis included 62 patients with a total of 86 implants (Fig. 1).

### Demographic characteristics

Most of the patients were non-smokers (73.68%) and 3.95% of the patients were former smokers. Another 9.21% of the patients smoked less than 10 cigarettes per day and 10.53% smoked more than 10 cigarettes per day. None of the patients smoked electronic cigarettes. Most of the patients (84.21%) displayed no periodontal bone resorption. Signs of previous periodontitis were observed in nine of the 76 patients (11.84%), although no active clinical manifestations were present at the time of surgery. A total of 65.9% of the patients reported no allergies, while a few patients had allergies to materials such as titanium and nickel (13.16%) or to medications primarily antibiotics (17.11%).

### Implant placement

Most patients (65.79%) received one single implant. 46.53% of the implants were placed in the upper jaw, with 8.91% of these located in the anterior zone. A crown restoration was planned in 89.11% of the cases, while bridge or removable prosthetic restorations were planned in seven cases. Most of the implants placed had a diameter of 4.1 mm (55.45%) and a length of 10 mm (58.42%). 97.03% of the implants were placed with a terminal insertion torque greater than 20Ncm. No complication such as injuries, damages to neighbouring teeth, infection, bleeding or swelling was reported. Additionally, no SADE were reported. Only one technical complication was reported as an insufficient tray holder (poor fit) which led to procedural difficulties and unreliable marking. Overall, two device-related adverse events occurred in two patients (2.63%). Both cases reported were trismus where the patients could not open their mouths sufficiently during the procedure. The relation to the study device might be explained by the effect of tray positioning in the patient’s mouth. One non-device-related event occurred in one patient (1.32%), where the planned implant diameter could not be used due to insufficient primary stability. Instead, a wider implant was placed.

### Angular and spatial deviation

**Fig. 2:**
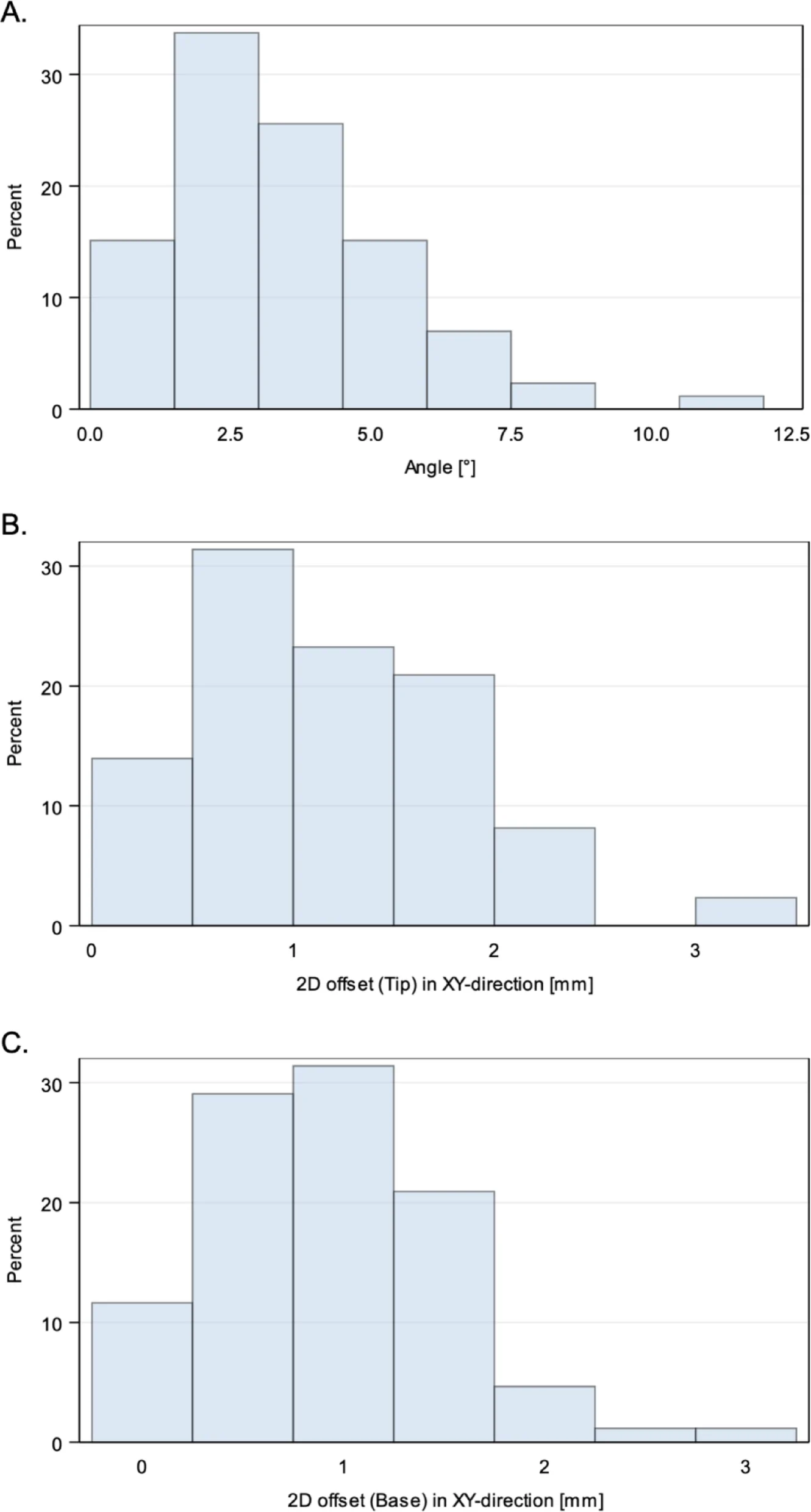
A. Histogram of the deviation of the largest angle from Z-axis (A), of the deviation on implant tip in XY-direction (B), of the deviation on the base in XY direction (C)

**Fig. 3:**
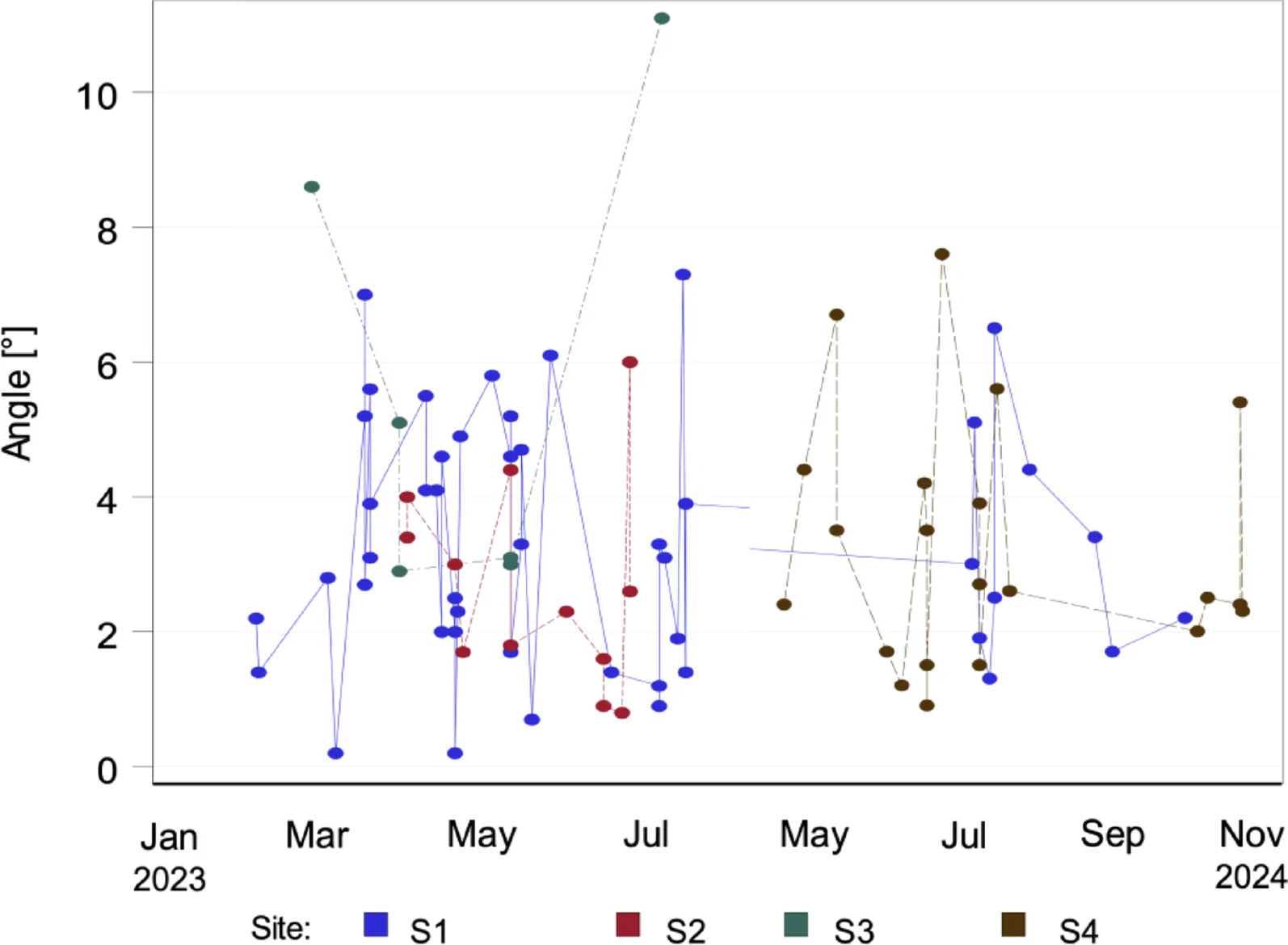
Spaghetti plot of the largest angle from the Z-axis over time for each site (S1 to S4)

**Fig. 4:**
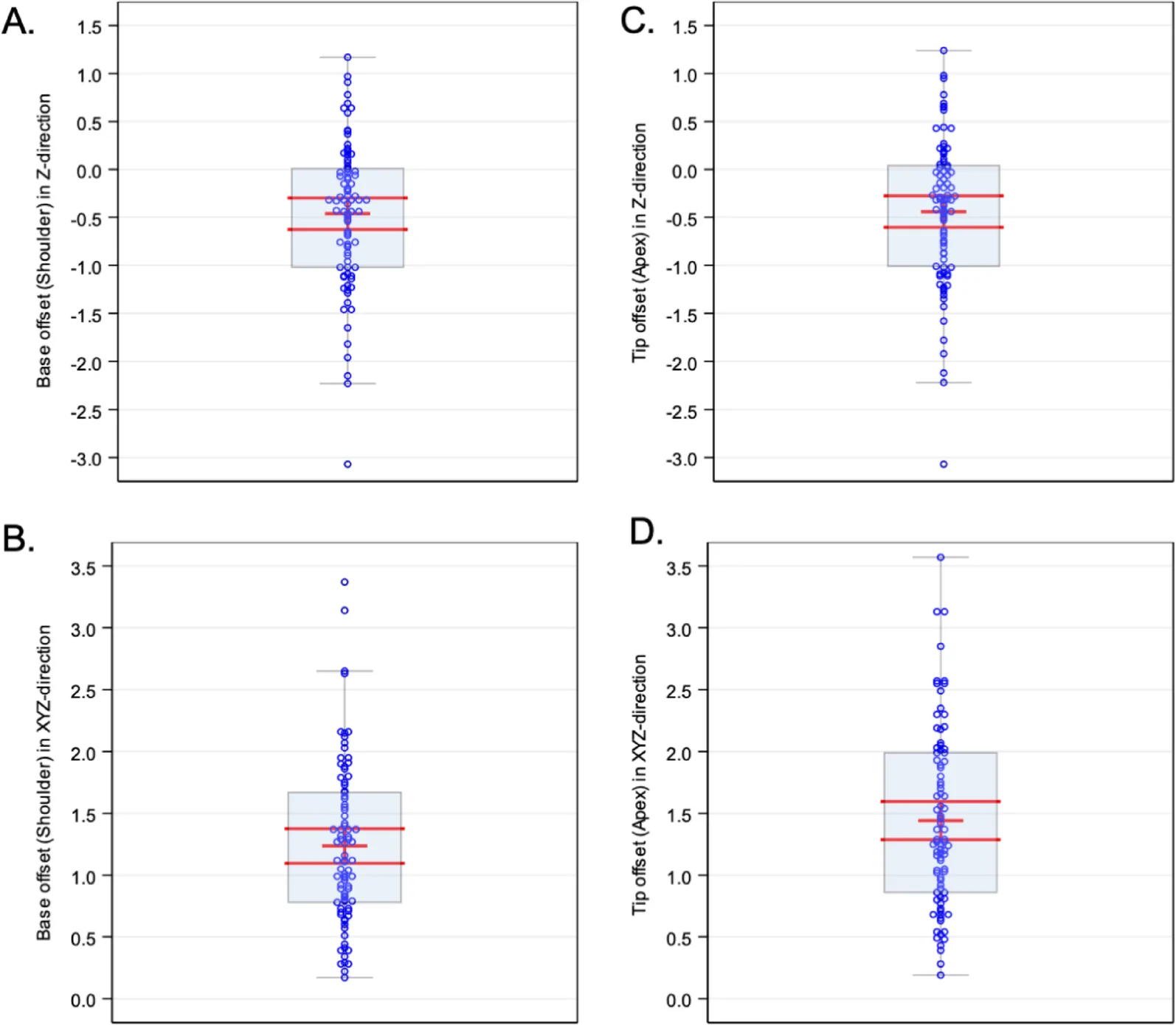
Scatter-Boxplot of apicocoronal deviation at implant base and tip (A and C, respectively) as well as the 3D deviation at implant base and tip (B and D, respectively). Red lines exhibit the mean and 95%-confidence limits

The overall mean angular deviation was 3.37° ± 2.01°, ranging from 0.20° to 11.10° (Figs. 2 and 3; Table 1). Details on the the largest angle from the Z-axis for each site is represented in the Fig. 3. The mean mesiodistal and orovestibular deviation in the XY-direction at implant tip was 1.18 ± 0.66 (range of 0.10–3.41 mm), and at the implant base 0.91 ± 0.58 mm (range 0.02–3.18 mm). The apicocoronal deviation in the Z-direction at the implant base showed a mean ± SD of -0.47 ± 0.77 mm, with a range of -3.07 to 1.17 mm and a 95% confidence interval (CI) of -0.63 to -0.3 mm. Similarly, apicocoronal deviation at implant tip was − 0.44 ± 0.76 mm, ranging from − 3.07 to 1.24 mm, with a 95% CI of -0.60 to -0.28 (Fig. 3). Furthermore, the 3-dimensional (3D) mesiodistal, orovestibular, and apicocoronal deviation in the XYZ-direction showed a mean ± SD of 1.24 ± 0.65 mm at the implant base and 1.4 ± 0.72 mm at the implant tip, with ranges of 0.17 to 3.37 mm and 0.19 to 3.57 mm, respectively. The corresponding 95% CIs were 1.10–1.38 mm (implant base) and 1.29–1.6 mm (implant tip) (Fig. 4).

**Table 1:** Mean values and their standard deviation (SD), as well as minimum and maximum values of angular deviation and spatial deviations

Angle (°)	*n*	min	max	mean	SD
	86	0,20	11.10	3.37	2.01
mesiodistal and orovestibular deviation (tip- mm)	86	0.10	3.41	1.18	0.66
mesiodistal and orovestibular deviation (base - mm)	86	0.02	3.18	0.91	0.58
Apicocoronal deviation (tip– mm)	86	-3.07	1.24	-0.44	0.76
Apicocoronal deviation (base – mm)	86	-3.07	1.17	-0.46	0.77
3D deviation (tip - mm)	86	0.19	3.57	1.44	0.72
3D deviation (base - mm)	86	0.17	3.37	1.24	0.65

### Performance evaluation

**Fig. 5:**
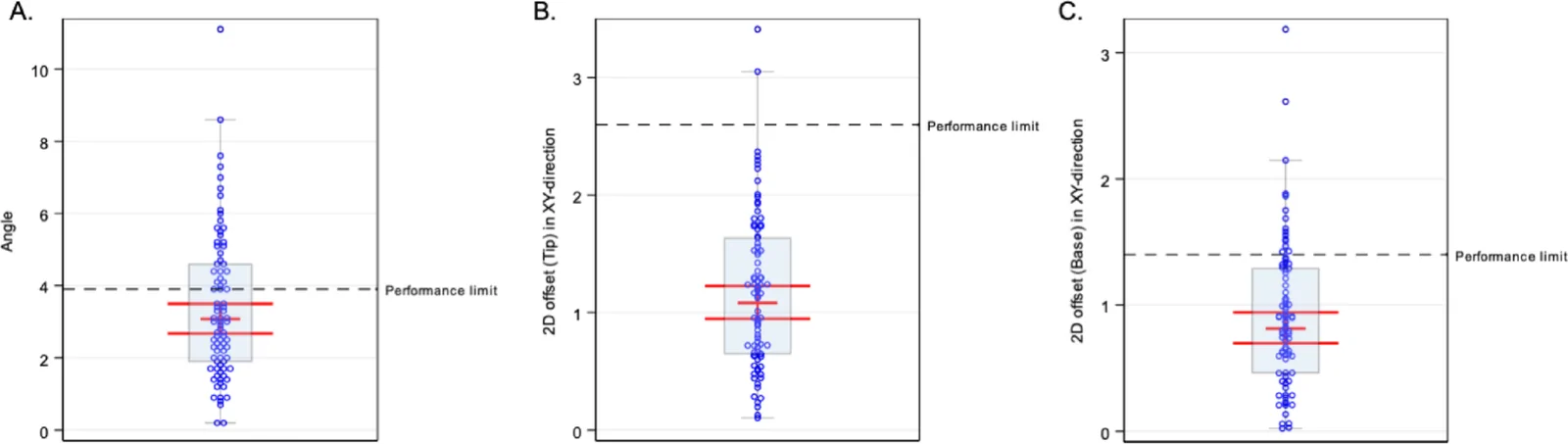
Scatter-Boxplot of angle deviation (A), of mesiodistal and and orovestibular deviation at implant tip and base (B and C, respectively). Red lines exhibit the mean and 95%-confidence limits. The performance limit is represented by the dashed line

As presented in Table 2, as per the statistical analysis, the mean of the square root transformed deviation of the largest angle from the Z-axis was 1.75 sqrt degrees, which was significantly better than the performance goal of 1.97 sqrt degrees. Additionally, the mean of the square root transformed, mesiodistal, and orovestibular deviation on the implant tip in the XY direction was 1.04 sqrt mm, which was significantly better than the performance goal of 1.61 sqrt mm. After square root transformation, the mean mesiodistal and orovestibular deviation at the implant base in the XY direction was 0.90 sqrt mm, representing a statistically significant difference compared with the performance goal of 1.18 sqrt mm. Scatter-box plots of the angular and spatial deviations are presented in Fig. 5 with mean values, confidence limit, and the set performance limit.

**Table 2:** Statistical analysis of the trueness regarding the angular deviation, the mesiodistal and orovestibular deviation at implant base and tip (square-root-transformed)

Angular deviation (°)	*n*	Mean vs. performance goal	Mean difference (upper 95%-CL)	*p* value
	86	1.75 vs. 1.97	-0.22 (-0.10)	< 0.001
Mesiodistal and orovestibular deviation (tip- mm)	86	1.04 vs. 1.61	-0.57 (-0.50)	< 0.001
Mesiodistal and orovestibular deviation (base - mm)	86	0.90 vs. 1.18	-0.28 (-0.21)	< 0.001

CL: confidence limits

### Procedure duration

The mean total duration of the procedure was 49.5 ± 25.40 min (mean ± SD), with a wide range varying from 6 to 120 min and 43.1 to 56.0 min as 95% confidence interval of the mean.

### Surgeon satisfaction outcome measures

**Fig. 6:**
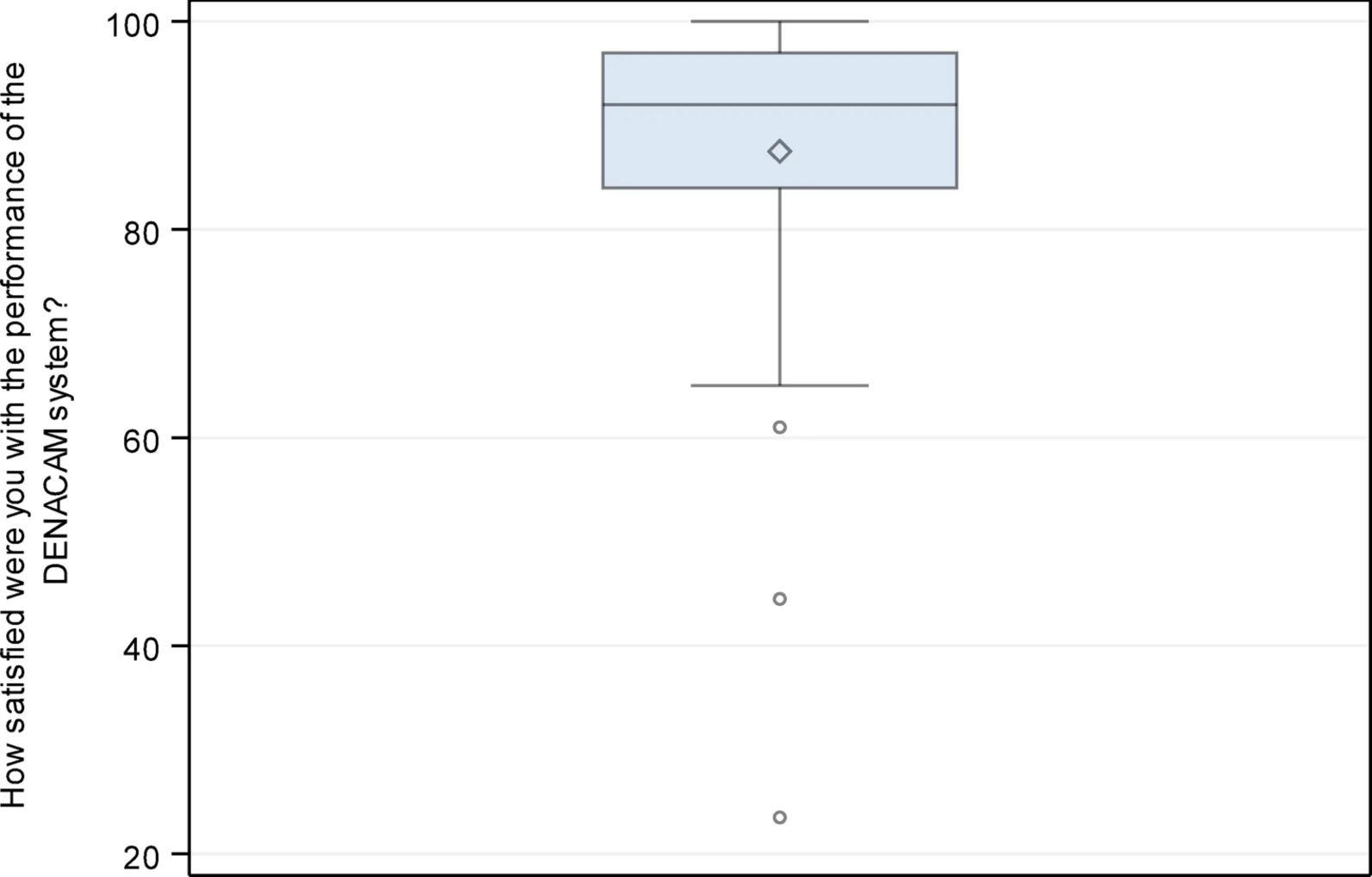
Boxplot of the satisfaction of the surgeon as measured using VAS

The recorded VAS ranged widely from 23.5 to 100.0 with a mean ± SD of 87.5 ± 14.3 and 83.9 to 91.1 as the confidence interval of the mean. A scatter box plot of the satisfaction of the surgeons is presented in Fig. 6.

## Discussion

The present prospective clinical trial shows high trueness, safety, and surgeon satisfaction in dental implant placement with the study-specific DNS. The system significantly reduced angular and positional deviations once compared to data extracted from benchmarks obtained with the freehand method supporting its trueness.

These findings align with previous studies investigating d-CAIS in clinical use. Yang and Geng (2024), which demonstrated higher implant trueness with immediate implant placement in the posterior maxillary region using d-CAIS compared to freehand surgery in 60 patients [27]. Comparable outcomes were reported in multiple randomized clinical trials, including one involving 34 patients undergoing single implant placement in the anterior maxilla [28] and another with 30 patients requiring implant placement in partially edentulous maxillae [10]. Aydemir & Arısan (2020) reported improved trueness of implant placement using DNS compared to freehand surgery in a split-mouth randomized controlled clinical trial [29]. For zygomatic implant placement, dynamic navigation technology showed better predictability in terms of trueness and precision compared to freehand surgery [30]. However, direct comparison between studies is challenging because different DNS are employed, each with its own hardware configuration, marker implementation, tracking technology, and calibration/registration protocols. These variations can influence also the user experience, making it difficult to generalize findings or establish a standardized benchmark across clinical trials. The present study evaluates the DENACAM Navigation system (MiniNavident, Liestal, Switzerland) which was already investigated previously [7, 16, 31]. In the present study, the mean angular deviation was in line with values reported clinically [9, 18] and exceeded slightly the values previously reported in vitro, which were 2.26°(± 1.87°) [7], 2.22°(± 1.54) [32] and 2.88°(± 2.03) [16] while remaining consistent with the findings of Taheri Otaghsara et al. 2023 [31]. This discrepancy can be attributed to the additional challenges encountered in vivo within a clinical setting, such as patient movement, limited intraoral accessibility, variations in bone quality, and the presence of soft tissues, all of which may reduce the precision achievable under controlled laboratory conditions [33]. In the present prospective cohort study, the minimum and maximum angular deviation was 0.20° and 11.10°, respectively. The maximum angular deviation was considerable, suggesting a learning-curve effect, indicating that the final implant position did not accurately align with the virtual plan. Higher deviation values may be clinically relevant, as they could increase the risk of inaccurate implant positioning, compromise prosthetic and esthetic outcomes, or injury to adjacent anatomical vital structures, particularly in difficult cases. As already mentioned by Tahmaseb et al. (2014) in case of CAIS, an accumulation of errors in the digital workflow can also result in increased inaccuracies [33]. These inaccuracies may result from various sources, including errors in intraoral scanning, matching between CBCT data and IOS, software planning, tray design and 3D printing, as well as handling during the clinical procedure, thus highlighting the importance of controls during surgery. Additionally, it could be shown, that the trueness of half-guided implant surgery, when only the implant bed preparation is performed with CAIS, is comparable with full guided CAIS [34]. Therefore, because the implants are placed freehand in a computer-assisted osteotomy using the present DNS, every step of the process - including osteotomy preparation - had the potential to introduce and accumulate errors. A comparable maximum angular deviation was reported by Aydemir & Arisan (2020) in a split-mouth randomized clinical trial on d-CAIS [29]. Surgeons using DNS in case of d-CAIS need to be aware of these possible deviations resulting in possible accumulation during the workflow. An advantage of d-CAIS over s-CAIS is the direct visualization of the surgical field and surrounding structures, allowing the clinician to adjust implant angulation during insertion if necessary. Generally, while the implant trueness is enhanced, the use of d-CAIS still needs safety margin of at least 2 mm to prevent any harm to anatomical structures as any s-CAIS device [35]. Safety was evaluated based on intraoperative and postoperative events, as well as the reporting of SADE. Long-term follow-up was not considered mandatory, as the DNS is a supportive technology. Therefore, any potential risks associated with its use are limited to the intraoperative and immediate postoperative period. Consequently, short-term safety assessment is sufficient to adequately evaluate its safety profile.

Objective performance goals were defined based on a comprehensive review of clinical studies reporting deviations in freehand implant placement. When compared to these benchmarks, the investigated system clearly demonstrated superior trueness across parameters, including the angular, mesiodistal and orovestibular deviations at implant base and tip. The magnitude of deviation observed with the system remained consistently below the performance goals reported for freehand surgery, thereby confirming its clinical benefit in terms of trueness. Exceeding these performance goals is clinically relevant, as smaller deviations contribute to accurate prosthetically driven implant placement, reduced risk of damage to adjacent anatomical structures, and improved long-term functional and esthetic outcomes [35]. Although defining performance goals provides a structured framework for the objective evaluation and comparison of different dynamic navigation systems (DNS), this approach represents a limitation, as no randomized control group was included. Therefore, an important limitation of this study is that the results were compared with literature-based benchmark data instead of a direct control group. Because the compared studies may differ in several parameters as patient populations or implant protocols, these indirect comparisons may introduce bias and reduce the reliability of the conclusions. Therefore, these related findings should be interpreted with caution.

Surgeon satisfaction was high, with mean VAS values of 87.5 ± 14.3 attributed to an intuitive workflow and intraoperative guidance. The VAS values exhibited a wide range primarily due to acclimatization to the DNS, which also reflects a learning-curve effect. High user acceptance is a key factor for successful clinical integration, as incorporating DNS into clinical practice often involves increased costs. Moreover, these systems have steep learning curves, which even being short, require consistent and repeated use to achieve proficiency [36]. Regarding the procedure duration, the total duration of the procedure was 49.5 ± 25.40 min (mean ± SD), with a wide range varying from 6 to 120 min. A direct comparison with freehand surgery is difficult to do as any clinical studies specifically investigating the clinical procedure duration of freehand implant placement. In fact, freehand surgery is considered as a conventional standard, and its timing is often viewed as routine and highly operator-dependent.

The present study has some limitations that should be addressed. The implant placement was non-guided after navigated osteotomy preparation due to the inability of the DNS to support navigated implant insertion. Additionally, while the system significantly reduced deviations compared to freehand benchmarks, direct randomized controlled comparisons would provide stronger evidence of superiority [37]. Although randomized controlled trials would offer the highest level of scientific evidence, this prospective clinical study includes a larger patient cohort compared to the previously mentioned clinical investigations. By being conducted as a multi-center study in four centres in Germany, potential biases related to socio-economic differences and patient selection were minimized. Only one surgeon had experience with the study-related DNS. The learning curve associated with adopting a new system should be considered when interpreting these findings, as it could explain the wide ranges for procedure duration and satisfaction. Another limitation of this study is the absence of patient-reported outcome measures, which restricts the evaluation of the clinical impact from the patient’s perspective, such as postoperative discomfort, satisfaction, and perceived treatment quality. Their inclusion in future studies would provide a more comprehensive assessment of patient-centered outcomes. This large cohort also included patients requiring between one and three implants in various tooth positions, allowing the results on the use of d-CAIS to be generalized. However, further clinical investigations need to be done. Cost-effectiveness analyses should also be considered to determine the practicality of routine implementation, particularly in resource-limited settings. Interestingly, two device-related adverse events were observed in two patients, both presenting with trismus that prevented adequate mouth opening during the procedure. This issue was not directly related to the study-specific DNS, as the hand piece used is comparable to that in freehand surgery, with similar interocclusal distance requirements. Tray positioning or dimensions appear to be the primary factors explaining the dropout of these two patients, which raises questions regarding the surgeons’ proficiency and experience with the DNS. Further research warrant to investigate the clinical impact of surgeon proficiency on implant trueness when using the DNS. This cohort was restricted to partially edentulous cases with sufficient teeth for marker fixation. Further research needs also to be focused on fully edentulous cases to highlight the trueness of DNS in other clinical contexts. In the present study, six patients (with 7 implants) dropped out before the operation began based on the investigators’ decisions due to including poor tray fit, unavailability of planning data at the start of surgery, delayed implant delivery, interference of the contra-angle piece between the sensor and DNS, restricted mouth opening, and missing calibration of the implant motor. These dropped outs are not incidental but reflect system-dependent workflow failures inherent to the real-world application of d-CAIS. Consequently, restricting the analysis to technically successful procedures may have led to an introduction of attrition bias.

## Conclusions

The present multi-center prospective clinical trial confirms the trueness of d-CAIS and highlights the intraoperative safety of the DNS. Surgeons reported general satisfaction with the use of the DNS. Future research should aim to validate these findings in larger, randomized cohorts and further investigate the performance differences among various DNS systems, as well as the influence of surgeon proficiency on implant placement trueness when using DNS.

## Supplementary Information

Below is the link to the electronic supplementary material.

Supplementary Material 1

## Data Availability

The original data supporting the findings of this study is available from the corresponding author upon reasonable request.

## Abbreviations

CAD/CAM: Computer-aided design and computer-aided manufacturing
CBCT: Cone-beam computed tomography
d-CAIS: Dynamic computer-assisted implant surgery
DNS: Dynamic navigation system
IOS: IntraOral scans
s-CAIS: Static computer-assisted implant surgery
SADE: Serious adverse device effects
SAE: Serious adverse effects
